# Relationships Between Theoretically Derived Short-Term Outcomes and Support for Policy Among the Public and Decision-Makers

**DOI:** 10.5888/pcd15.170288

**Published:** 2018-05-17

**Authors:** Carol L. Schmitt, Laurel Curry, Vanessa Boudewyns, Pamela A. Williams, LaShawn Glasgow, Deanna Van Hersh, Jeffrey Willett, Todd Rogers

**Affiliations:** 1 RTI International, Research Triangle Park, North Carolina; 2 Kansas Health Foundation, Wichita, Kansas; 3 Schroeder Institute at Truth Initiative, Washington, District of Columbia

## Abstract

**Purpose and Objectives:**

Policy change is a lengthy and complex process. Thus, it is important to articulate hypothesized causal pathways between advocacy activities and policy change outcomes and to identify and monitor early indicators of progress toward policy change.

**Intervention Approach:**

The Kansas Health Foundation supports grantee efforts to address the public health effects of obesity through evidence-based policy, systems, and environmental change interventions. To build support for policy, systems, and environmental changes in schools, workplaces, and health care and retail settings, grantees mobilize communities, educate government policy makers, and advocate with organizational decision makers.

**Evaluation Methods:**

To understand whether early outcomes from obesity-prevention advocacy efforts predict interim outcomes related to eventual policy change, we conducted surveys of the general public and of opinion leaders in Kansas, which were designed to measure components of Kansas Health Foundation’s theory of change. We then used structural equation modeling to test the theory of change’s underlying relationships by using support for obesity prevention policies as the outcome.

**Results:**

Our findings supported the hypothesized model: perceptions of obesity as a serious community problem influence beliefs about causes of the problem. Beliefs about causes predict beliefs about who is responsible for the solution to the problem, which in turn predicts support for obesity prevention policies.

**Implications for Public Health:**

Evaluators of advocacy for policy change interventions can use this approach to monitor proximal changes in public and opinion leader beliefs related to eventual policy change and to determine whether efforts are likely to be successful or need to be adapted or abandoned.

## Purpose and Objectives

More than one third of Americans have obesity (1), and implementing policies that change the environment in ways that promote healthy eating and physical activity are an important component of addressing obesity (2). However, public health programs cannot directly change policies; rather, they make a long-term investment in educating policy makers about the need for and value of policies and they work to build demand for policy change among community members (3).

Reaching an obesity policy goal may require many years of sustained effort, and developing interim measures of progress toward that goal provides funders with early indicators of success and an opportunity to refine and improve activities if expected milestones are not reached (4). Developing a theory of change is part of this process; the theory illustrates the hypothesized relationships between a program’s inputs, activities, outputs, and outcomes (5,6) and it serves as the basis for interim indicators of progress. Our study illustrates how testing a theory of change using structural equation modeling (SEM) methods — an analytic method for studying theory-based associations consistent with other examples in the literature (7) — can increase confidence in the hypothesized relationships between short-term policy advocacy outcomes and intermediate-term outcomes in the context of obesity prevention. Specifically, we test whether the short-term outcomes in our theory of change predict support for obesity-related policies, with the assumption that increasing support for obesity policies will lead to policy change, given sufficiently supportive contextual factors.

## Intervention Approach

In 2009, the Behavioral Risk Factor Surveillance System (BRFSS) indicated that 28.8% of Kansas adults had obesity. In response to this and other health trends such as low fruit and vegetable consumption and high rates of diabetes and physical inactivity, the Kansas Health Foundation established a Healthy Living Focus Area. The foundation supported Healthy Living Focus Area grantee efforts in Kansas to address obesity and related chronic diseases through traditional health behavior change programs and policy, systems, and environmental change interventions. Policy, systems, and environmental change interventions are population-based public health approaches designed to make healthy choices — in this case, active living and healthy eating — the easy choices (8). The approaches include adoption of policies that create an environment favorable to regular exercise and access to healthy foods.

Healthy Living Focus Area policy, systems, and environmental change efforts were based on Institute of Medicine recommendations for reducing obesity (9) and input of leading experts in the area. As a result, Kansas Health Foundation funded grantee efforts to develop, build support for, and in some cases implement systems change and policies in diverse settings. For example, some grantees promoted and supported policies and curriculum changes focused on physical activity and nutrition in schools and child care facilities. Other grantees focused on local policies to improve the built environment and make healthy foods more affordable and accessible. To build support for these changes, grantees educated and mobilized community members, educated government policy makers, and advocated with organizational decision makers.

## Evaluation Methods

### Theory of change development

As part of a comprehensive Healthy Living Focus Area evaluation, Kansas Health Foundation staff and external evaluators collaboratively developed a theory of change (10). The theory of change was developed through a combination of deductive (drawing on scholarly theory) and user-focused (guiding intended users in the articulation of their program theory) approaches (11). The resulting theory of change consisted of 2 pathways that reflect the broad types of Healthy Living Focus Area grant initiatives: a traditional public health pathway, in which healthy eating and active living outcomes are achieved through evidence-based initiatives to change health behavior, and a policy, systems, and environmental change pathway, in which such outcomes are achieved through policy advocacy and systems change approaches. The latter pathway was adapted from the Advocacy and Policy Change Composite Logic Model and the Visual Framework of Public Policy Strategies (12,13). In this article, we focus on our use of SEM methods to test the presumed links between the short-term outcomes expected to occur as a result of planned grantee activities — changes in perceptions about obesity and its policy solutions — and the longer-term-outcomes associated with eventual policy change in the policy, systems, and environmental change pathway — support for obesity policies. Data were collected to assess these outcomes at the state level and not specifically linked to grantees’ activities, which were primarily at the local level.

### Theory of change testing

We tested the hypothesized interrelationships in the theory of change between perceived seriousness of the obesity problem, beliefs surrounding the problem, and support for policies related to obesity using SEM. By testing the model fit — the extent to which the data collected support the hypothesized relationships in the theory of change — we can determine the extent to which early outcome indicators are useful measures of progress toward eventual support for obesity policies. If these relationships are supported, program implementers and evaluators working on building support for obesity policies can use this theory of change model to monitor proximal changes in public and opinion leader beliefs that are related to policy support and eventual policy change.

### Operationalizing key constructs for SEM

**Figure 1:**
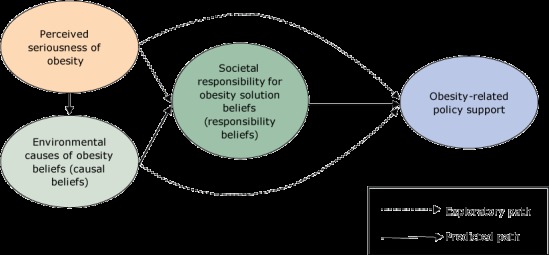
Predicted theory of change model used to operationally define the constructs of the Healthy Living Focus Area theory of change, the relationships between these constructs, and the hypothesized relationships of the constructs to support for obesity policies, Kansas Health Foundation, 2014. Dashed lines represent exploratory paths not predicted in the theory of change. Solid lines are paths predicted in the theory of change.

We hypothesized that, when implemented, grantee educational and advocacy activities would lead to increased perceived seriousness of obesity in communities and eventually to increased support for policy changes to address it. Figure 1 describes how we operationally defined the constructs of the theory of change, the relationships between these constructs, and hypothesized relationships of the constructs to support for obesity policies. These relationships are based, in part, on the Health Belief model (14), which posits that beliefs about the seriousness of a problem can be used to predict behaviors. It is also based on the theory of perceived responsibility and social motivation, which postulates that there is an association between causal attributions and support for government action to reduce a social problem (15,16). This theory proposes that causal attributions of a social problem influence solution attributions, which shape personal behaviors and support for solutions to address the problem.

### Data collection

We conducted 2 cross-sectional telephone surveys during the summer of 2014: 1 among the Kansas public (the General Public Survey) and the other among opinion leaders in Kansas (the Opinion Leader Survey). RTI International’s institutional review board approved all study procedures. The surveys took approximately 15 minutes to complete. Respondents were not provided material incentives.

The General Public Survey was a representative household survey of 2,203 Kansas residents aged 18 years or older conducted from May 12 to August 4, 2014. Because the prevalence of cellular telephone–only households has increased (17), and to maximize response rates, we used a dual-frame telephone survey with an overlap design. The sampling frame consisted of all landline and cellular telephone numbers in Kansas. On the landline frame, 1 adult was randomly selected in each household reached; on the cellular telephone frame, the person who answered the phone was selected. Sample weights were created to map the sample to the Kansas population. The overall response rate by using the American Association for Public Opinion Research 3 formula (18) was 15.2%, which is consistent with the rates achieved in similar population surveys (19).

The Opinion Leader Survey was a 2-stage probability telephone survey conducted from May 12 to September 12, 2014. In the first sampling stage, we selected 73 of 105 Kansas counties proportional to the eligible population with probability minimum replacement (20). To identify participants in the 73 counties, we created a sampling frame representing opinion leaders in 9 sectors (business; education; city, county, state, and federal officials; ethnic and health organizations; and media), based in part on previous studies of opinion leaders (19,21) and consultation with Kansas Health Foundation. We found contact information (names, telephone numbers, and email, where possible) using publicly available information gathered through web searches, such as searches of Kansas state and county websites.

In the second sampling stage, we stratified by urban/rural designation to obtain estimates as close to representative of the population distribution of urban and rural counties as possible. Urban counties were defined as large fringe metropolitan, medium metropolitan, and small metropolitan areas, whereas rural counties were defined as micropolitan and noncore (22). We also stratified by office type within sectors (eg, the education sector had 2 office types: superintendents and school board presidents). For office types with small sampling frame sizes (n < 67) we selected the entire frame. For office types with larger frame sizes, we selected a simple random sample (n = 67) with near equal numbers of leaders from rural and urban counties to yield the number of responses necessary for an adequately powered study, aiming for 1,650 as our sample size. On the basis of surveys of opinion leaders in other states, where we have had response rates ranging from 55% to 65%, we calculated that we would need a total sample of 1,650 for a yield of at least 900 completes (needed to keep the margin of error at a reasonable level). Our total sample size was 1,654, and of those, 912 completed the survey. Before calling opinion leaders, we sent them lead letters explaining the study purpose, study sponsor, and survey duration and informing them that they would receive a telephone call within the next few days from an interviewer.

The overall response rate was 55%. Response rates varied by position type, with the highest rate among health organizations (90.6%) and the lowest rate among state senate members (30.8%) and members of Congress (0).

### Sample

General Public Survey and Opinion Leader Survey participant characteristics are detailed in Table 1, along with demographics from the 2013 Kansas census of the population aged 18 or older. The opinion leader sample contained a higher proportion of men, adults aged 45 or older, participants representing rural areas, whites, and people identifying as conservative. The difference in geographic distribution between General Public Survey and Opinion Leader Survey respondents is a result of the difference in study design; the General Public Survey is an adult population study (and the largest proportion of Kansans live in urban areas), whereas the Opinion Leader Survey is based on leaders in counties (and our sample was designed to ensure equal representation of urban and rural counties). Demographic characteristics of the General Public Survey and census populations were very similar.

**Table 1:** Demographic Characteristics of the Kansas General Public Survey and Opinion Leader Survey Samples, 2014

Characteristic	General Public Survey, Weighted %	Opinion Leader Survey, Weighted %	Census, Kansas %^a^
**Sex**			
Male	50.7	57.0	50.7
Female	49.3	43.0	49.3
**Age, y**			
18–24	13.6	8.1	14.1
25–44	34.0	12.3	33.8
45–64	33.7	55.3	33.6
≥65	18.7	24.3	18.5
**Geographic distribution^b^**			
Urban	65.0	28.7	NA
Rural	35.0	71.3	
**Race/ethnicity**			
White (non-Hispanic)	80.4	93.7	80.2
Black (non-Hispanic)	5.7	0.8	6.0
Hispanic	9.2	1.0	9.1
Other (non-Hispanic)^c^	4.8	4.4	4.6
**Education attainment**			
None or less than high school diploma	11.6	NA	11.8
High school diploma or general equivalency diploma	30.2		30.2
Some college	28.6		28.5
College graduate	29.6		29.5
**Annual household income, $**			
<25,000	26.2	NA	NA
25,000–49,900	32.7		
50,000–74,900	23.2		
≥75,000	18.0		
**Political philosophy^d^**			
Conservative	42.1	51.3	NA
Moderate	35.4	39.0	
Liberal	22.5	9.7	
**Overweight or at risk for being overweight^e^**			
No	61.7	66.3	NA
Yes	38.3	33.7	

Abbreviation: NA, not available.

a Using data from 2013 US Census: http://www.census.gov/popest/data/state/asrh/2013/SC-EST2013-ALLDATA5.html.

b The difference in geographic distribution between General Public Survey and Opinion Leader Survey respondents is a result of the difference in study design; the General Public Survey is an adult population study (and the largest proportion of Kansans live in urban areas), whereas the Opinion Leader Survey is based on leaders in counties (and our sample was designed to ensure equal representation of urban and rural counties).

c Asian, Native Hawaiian or other Pacific Islander, American Indian or Alaska Native, and multiracial.

d Conservative = answered “very conservative” or “somewhat conservative,” moderate = answered “moderate — neither conservative nor liberal,” and liberal = answered “somewhat liberal” or “very liberal” in response to “How would you describe your overall political philosophy?”

e Answered yes to “Has a health care professional ever told you or someone in your household that you are overweight or at risk for being overweight?”

### Measures

We developed items to measure the constructs in the theory of change. The items in the General Public Survey and Opinion Leader Survey were designed to be directly comparable to one another. To develop the survey items, we used a literature review of similar studies, the theory of change, investigator consensus, and survey methodologist feedback. We also conducted cognitive testing by using think-aloud methods with a small convenience sample of 5 Kansas opinion leaders nominated by Kansas Health Foundation and 5 members of the Kansas general public. We created variables representing components of the theory of change by combining participants’ responses to various closed-ended items. For all scales, coefficient H was used as a measure of an item’s construct reliability using the weighted data (23).

Four items were used to assess participants’ perceptions of the seriousness of the obesity problem and its antecedents in their community (ie, obesity and overweight among children, obesity and overweight among adults, lack of regular exercise, and unhealthy eating). Responses were rated on a 3-point scale (1 = not at all a problem, 2 = somewhat serious problem, 3 = a very serious problem). Items were averaged to form a scale (General Public Survey: coefficient H = 0.79, mean [standard error (SE)], 2.38 [0.02]; Opinion Leader Survey: coefficient H = 0.81, mean [SE], 2.14, [0.06]). Higher scores indicate greater perceptions that obesity and related factors, namely exercise and unhealthy eating, are a serious problem in the community.

By environmental causes of obesity, we mean the structural, systemic, and informational factors that influence individuals’ decisions to consume certain foods over others and to engage in some activities over others (eg, more or less physically active choices). To measure the extent of participants’ beliefs that one can attribute the causes of obesity to environmental factors, participants responded to various items asking the extent to which each item was a reason behind the obesity issue (“healthy foods are expensive,” “unhealthy food is inexpensive and easy to find,” “there are too many unhealthy foods, snacks, and drinks available in schools,” “there are not enough safe places for people to be physically active indoors,” and “people don’t have enough information about the nutrition and calories in their food”). Items were adapted from a published national survey (24). Responses were measured on a 3-point scale (0 = not a reason at all, 1 = minor reason, 2 = major reason). The 5 items were summed to form an index of causal beliefs; higher values represent stronger beliefs in environmental causes of obesity (range, 0–10) (General Public Survey mean [SE], 5.75 [0.07]; Opinion Leader Survey mean [SE], 5.75 [0.07]).

Participants rated 5 groups on their level of responsibility for solving the obesity problem in Kansas: “the food industry,” “the U.S. federal government,” “state and local governments,” “employers,” and “schools.” Participants were asked to rate the level of responsibility for solving the obesity problem in Kansas for each group: 1 = little of the responsibility, 2 = some of the responsibility, or 3 = most of the responsibility. Items were averaged to form a scale (General Public Survey: coefficient H = 0.81, mean [SE], 1.79 [0.01]; Opinion Leader Survey: coefficient H = 0.84, mean [SE], 1.66 [0.04]). Higher scores corresponded with beliefs that more responsibility should be placed on societal entities.

We measured participants’ support for obesity-related policy change by using a 7-item measure on which participants were asked, “What is your opinion about a policy that would . . . 1) require more physical activity in schools; 2) place a tax on the sale of sugar-sweetened beverages; 3) eliminate sales tax on fresh fruits and vegetables; 4) provide state funding for low-income schools to purchase fresh, local food; 5) limit the availability of unhealthy foods and beverages in schools; 6) fund bike paths, trails, sidewalks, and other projects to promote biking and walking; and 7) provide rewards to retailers that sell fresh, local foods.” Responses were based on a 5-point scale ranging from 1 = strongly against to 5 = strongly in favor. Items were averaged to form a scale, where higher scores represent greater support for obesity-related policies (General Public Survey: coefficient H = 0.75, mean [SE], 4.10 [0.02]; Opinion Leader Survey: coefficient H = 0.86, mean [SE] 3.83 [0.10]).

### Analysis

We used a structural model with observed variables to test the fit of our hypothesized model to the data using maximum likelihood estimation in the LISREL software version 9.2 (Scientific Software International, Inc). All variables were included as observed (manifest) variables in the model. We evaluated model fit first in terms of how well the overall model fit the observed data, as indicated by 2 fit indices: the χ^2^ test and a root mean square error of approximation [RMSEA] of less than .06 (25). A nonsignificant χ^2^ statistic is preferable because it reflects “exact fit” between the hypothesized model and the data; however, we rely primarily on the RMSEA because the χ^2^ is highly affected by sample size: larger samples produce larger χ^2^ results that are more likely to be significant (type I error). Once model fit was confirmed, we also examined the significance and strength of estimated parameters and the variance accounted for by variables. The same model was run for both the General Public Survey and Opinion Leader Survey by using weighted data. The models were then compared to see whether a similar pattern of effects was found.

## Results

Table 2 lists the results of the confirmatory factor analysis for each of the constructs. Most of the items were strongly associated with the factor, and all factor loadings were above the recommended cut off of 0.30 (26,27).

**Table 2:** Factor Loadings of the Kansas General Public Survey and Opinion Leader Survey Samples, 2014

Category	Opinion Leader Survey			General Public Survey		
	Factor Loading	Cronbach α	Coefficient H	Factor Loading	Cronbach α	Coefficient H
**Perceived seriousness of obesity**						
Obesity and overweight among adults	0.62	0.79	0.81	0.69	0.79	0.79
Obesity and overweight among children	0.70			0.68		
Lack of regular exercise	0.74			0.74		
Unhealthy eating	0.77			0.66		
**Societal responsibility for obesity solution (responsibility beliefs)**						
The food industry	0.56	0.75	0.84	0.49	0.74	0.81
The US federal government	0.76			0.78		
State and local governments	0.86			0.81		
Employers	0.49			0.49		
Schools	0.36			0.43		
**Obesity-related policy support**						
Require more physical activity in schools	0.65	0.75	0.86	0.53	0.72	0.75
Tax on the sale of sugar-sweetened beverages	0.48			0.44		
Eliminate sales tax on fresh fruits and vegetables	0.57			0.39		
State funding for low-income schools to purchase fresh, local food	0.70			0.66		
Limit the availability of unhealthy foods and beverages in schools	0.51			0.53		
Fund bike paths, trails, sidewalks, and other projects to promote biking and walking	0.76			0.59		
Reward retailers that sell fresh, local foods	0.80			0.56		

### General Public Survey results

**Figure 2:**
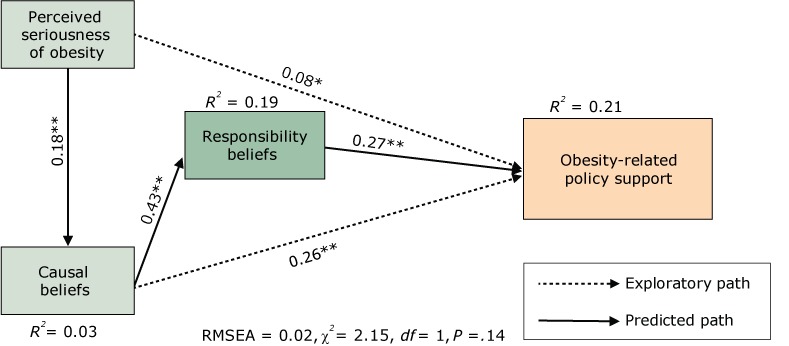
Mediational model for the General Public Survey sample, 2014. Abbreviations: *df*, degrees of freedom; RMSEA, root mean square error of approximation. *P < .01; **P < .001.

For the General Public Survey data, the hypothesized model met the criteria for good model fit (RMSEA = .02, χ^2^ = 2.15, *df* = 1, *P* = .14). All theory-predicted parameters (ie, the thick lines in Figure 2) were significant and with the expected sign (ie, the expected direction). In addition, 2 of the exploratory paths were also significant. Overall, the 3 constructs accounted for 21% of the variance in policy support. Greater perceived seriousness of obesity predicts greater environmental causal beliefs for why more people are becoming overweight and obese (β = 0.18, *P* < .001), and greater obesity-related policy support (β = 0.08, *P* < .01). Stronger environmental causal beliefs predicted greater beliefs that societal factors (eg, business and government) are responsible for solving the obesity problem (β = 0.43, *P* < .001). Stronger environmental causal beliefs were also associated with increased support for obesity-related policy change (β = 0.26, *P* < .001). Finally, stronger societal responsibility beliefs predicted greater obesity-related policy support (β = 0.27, *P* < .001).

We found a complex set of relationships between the variables in the model. Although perceived seriousness of obesity did not have a direct effect on societal responsibility beliefs, it did have a significant total effect (β_total_ = 0.08) (Table 3). The results suggest that beliefs about the causes of obesity explain the relationship between perceptions of the seriousness of obesity and beliefs about responsibility for addressing obesity. That is, perceiving obesity as a severe community problem was not directly related to beliefs that institutions (eg, government) should play a role in solving that problem; rather, perceiving obesity as a serious community problem was associated with attributing obesity to environmental causes, and these attributions were associated with assigning responsibility for its solution to societal institutions. The model suggests that beliefs about the causes of obesity have the strongest total effect on policy support (β_total_ = 0.37) (Table 3).

**Table 3:** Standardized Direct, Indirect, and Total Effects of Variables in the Theory of Change, Kansas General Public Survey, 2014 (n = 2,203), 2014

Variable	Perceived Seriousness of Obesity	Causal Beliefs	Responsibility Beliefs
**Causal beliefs**			
Direct	0.18^b^		
Indirect	—		
Total	0.18^b^		
**Responsibility beliefs**			
Direct	—	0.43^b^	
Indirect	0.08^a^	—	
Total	0.08^a^	0.43^b^	
**Policy support**			
Direct	0.08^a^	0.26^b^	0.27^b^
Indirect	0.07^b^	0.12^b^	—
Total	0.14^b^	0.37^b^	0.27^b^

—, No significant path between the variables.

a *P* < .01.

b *P* < .001.

### Opinion Leader Survey results

**Figure 3:**
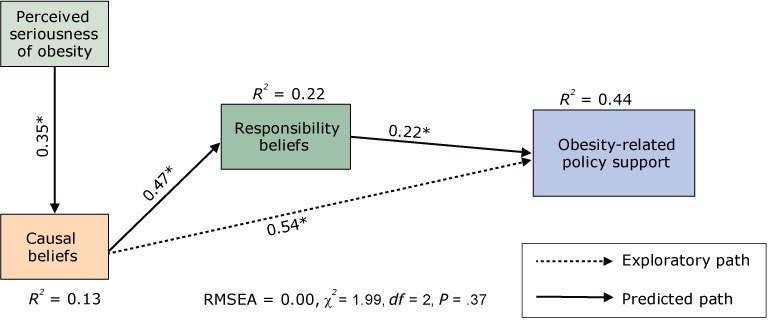
Mediational model for the Opinion Leader Survey sample, 2014. Abbreviations: *df*, degrees of freedom; RMSEA, root mean square error of approximation. *P < .001.

For the Opinion Leader Survey data, the hypothesized model also met the criteria for good fit (RMSEA = 0.00, χ^2^ = 1.99, *df* = 2, *P* = .37). As with the General Public Survey data, all theory-predicted parameters were significant, with the expected sign and show that the predicted relationships in the theory of change model hold for the public and opinion leaders (Figure 3). One difference in the pattern of results from the General Public Survey data was that, for the opinion leaders, perceived seriousness of obesity did not have a direct effect on policy support (this path was not predicted by the theory of change; instead, it was exploratory). Perceived seriousness of obesity had a significant indirect effect on policy support, with a combined total effect (β_total_ = 0.23) similar to the total effect of responsibility beliefs on policy support (β_total_ = 0.22). Overall, perceived seriousness of obesity, environmental causal beliefs, and societal responsibility beliefs accounted for 44% of the variance in policy support. Increases in perceived seriousness of obesity, environmental causal beliefs, and societal responsibility beliefs were associated with greater support for obesity prevention policies. Similar to the findings in the General Public Survey sample, findings in the Opinion Leader Survey showed that causal beliefs had the strongest total effect on policy support (β_total_ = 0.64) (Table 4). The effect of causal beliefs was not fully mediated by responsibility beliefs; instead, causal beliefs had direct and indirect effects on policy support.

**Table 4:** Standardized Direct, Indirect, and Total Effects of Variables in the Theory of Change, Kansas Opinion Leader Survey, 2014 (n = 912), 2014

Variable	Perceived Seriousness of Obesity	Causal Beliefs	Responsibility Beliefs
**Causal beliefs**			
Direct	0.35^b^		
Indirect	—		
Total	0.35^b^		
**Responsibility beliefs**			
Direct	—	0.47^c^	
Indirect	0.18^b^	—	
Total	0.16^b^	0.47^c^	
**Policy support**			
Direct	—	0.54^c^	0.22^b^
Indirect	0.27^a^	0.10^a^	—
Total	0.23^a^	0.64^c^	0.22^b^

—, No significant path between the variables.

a *P* < .05.

b *P* < .001.

c *P* < .01.

## Implications for Public Health

Healthy Living Focus Area grantees conducted education and community mobilization activities to promote evidence-based policies with the potential to decrease obesity. Grantees conducted these activities across the state and in diverse settings over a short period of time (3 years), although the foundation has committed to a longer-term investment in decreasing obesity in Kansas. The specific activities were as varied as the settings. In some cases, grantees advocated with school districts and individual schools for new policies or systems; in other cases, they worked with individual communities to plan and advocate for changes in the built environment that would better support physical activity. We developed our theory of change and associated indicators for the Healthy Living Focus Area evaluation but also as part of a longer-term plan to ensure that the foundation can document progress toward support for obesity policy change. This progress may occur because of activities conducted by former Healthy Living Focus Area grantees or because of future initiatives supported by the foundation or other funders.

In this study, we used SEM methods to test the logic underlying the policy, systems, and environmental change pathway in the Healthy Living Focus Area theory of change to document the extent to which our indicators of progress toward obesity policy changes predict support for obesity policies. We found that obesity-related beliefs held by the general public and opinion leaders in Kansas (short-term outcomes) predicted support for obesity policy change (longer-term outcomes) in the manner hypothesized by the theory of change. Under favorable circumstances, we would expect that organizations and local governments would adopt these policies if a large enough proportion of decision makers (including policy makers) and the public supported them.

Finding support for the pathways in the theory of change is an important contribution to the challenge of evaluating interventions focused on policy change. Tobacco control, considered the model for successful policy approaches to public health issues including obesity (28), overwhelmingly focuses on the impact of policies on behavior with little focus on how education and advocacy activities lead to those policy changes (29). A focus on how activities lead to support for policy is particularly important for the long-term investment needed to build support for obesity policies (3) and to ensure that intervention messages are relevant to the audiences targeted (30).

These findings have several implications for funders, program implementers, evaluators, and other stakeholders. We found that when both opinion leaders and the general public believe that obesity is a serious problem in their community, they are more likely to believe that the causes of obesity are at least in part environmental. In turn, consistent with theory, when opinion leaders and the general public perceive various environmental factors as contributing to obesity, they are more likely to hold organizations such as schools, government, and the food industry responsible for addressing it (16). Finally, opinion leaders and the public who believe that organizational bodies have a role in addressing obesity are more likely to support obesity policy change. Each of these belief sets represents progress toward policy support needed for evaluation of policy change interventions. Beliefs can be changed through interventions, including media campaigns. Policy change efforts, then, can include interventions that change opinion leader and public beliefs about the seriousness and causes of obesity and beliefs about who has the responsibility to address obesity.

However, the value of this approach (and the likelihood of finding significant results) is highly contingent on the care with which the beliefs measured are selected. This contingency applies to any analytic approach used to examine predictors of support for policy change. There is an extensive literature defining beliefs, attitudes, intentions, and behaviors, along with recommended approaches to identifying relevant constructs (31,32), on which one might draw and construct indicators and measures. Using theory to inform an evaluation theory of change provides the basis for generalizing and applying findings, while including a participatory component ensures that findings are relevant to the intervention you are evaluating. For example, consistent with the theory of reasoned action (32), our findings suggest public and opinion leader support for obesity policy change could be increased through interventions that strengthen beliefs about the seriousness and causes of obesity. They also suggest a role for changing normative beliefs about how much responsibility social institutions (eg, government, business) have for addressing obesity and could be further expanded to explore organizational beliefs. Conversely, a more participatory approach such as concept mapping (31) yields a theory of change very specific to the intervention being evaluated. In our study, the beliefs examined were specific to the Healthy Living Focus Area, and without that specificity, we would not have findings to inform the intervention we were evaluating.

Finally, careful consideration of the audience targeted for policy change is important, because, for example, conservatives may be persuaded by different arguments than liberals (33). The beliefs associated with support for the obesity policies we examined in this study were derived from a combination of theory, previous qualitative work we have conducted on beliefs and policy change (34), and interviews conducted with foundation staff responsible for the initiative. It was subsequently modified after we mapped the objectives of all Healthy Living Focus Area grantees to the preliminary theory of change (10).

Although our findings are promising and consistent with theory, we acknowledge several limitations. First, these data focus only on obesity-related beliefs and policy support, although we believe they have wider application to other public health policy change goals. Second, these data were generally representative at the state level, while most Healthy Living Focus Area grantee activities were conducted in local communities. As a result, we were unable to examine relationships between localized activities and outcomes measured at the state level. Third, respondents were the public and opinion leaders in Kansas, a state that ranks among the most politically conservative, with a strong commitment to small-government principles, and is in the Midwest, a region of the United States where obesity levels are among the highest. Thus, our findings may not be generalizable to the public and opinion leaders in other states. Fourth, our opinion leader sample consisted of multiple sectors believed to influence the policy process, including the media, business leaders, and local and state elected officials. Although all of these sectors can influence policy change, only a subset of them (ie, local and state elected officials) can propose and vote for a policy. Fifth, even with the additions of causal responsibility and environmental influence factors, the proposed theory of change linking advocacy activities to policy support is a simplistic representation of the complex policy development and implementation processes that typically involve various activities to manifest outcomes; this may challenge the utility of the theory of change in measuring early progress on multiple pathways to policy change. Finally, although the relationships depicted in the theory of change are presumed to be longitudinal, our data are cross-sectional. Follow-up longitudinal studies examining change over time may help solidify our understanding of the direction of the association and whether proximal variables (eg, causal beliefs) predict changes in distal outcomes (eg, policy support).

Limitations notwithstanding, our findings provide some evidence that the Healthy Living Focus Area theory of change is a useful framework for monitoring progress, informing course corrections, and increasing the chance that funder investments in the efforts of program implementers will lead to desired outcomes. As the evidence base grows for policy, systems, and environmental change approaches to chronic disease prevention and control, this work may serve as a model for funders and evaluators seeking to monitor and measure progress toward policy, systems, and environmental change goals.

## References

[1] Ogden CL , Carroll MD , Fryar CD , Flegal KM . Prevalence of obesity among adults and youth: United States, 2011–2014. NCHS Data Brief 2015;(219):1–8. 26633046

[2] Gortmaker SL , Swinburn BA , Levy D , Carter R , Mabry PL , Finegood DT , Changing the future of obesity: science, policy, and action. Lancet 2011;378(9793):838–47. 10.1016/S0140-6736(11)60815-5 21872752 PMC3417037

[3] Huang TTK , Cawley JH , Ashe M , Costa SA , Frerichs LM , Zwicker L , Mobilisation of public support for policy actions to prevent obesity. Lancet 2015;385(9985):2422–31. 10.1016/S0140-6736(14)61743-8 25703113

[4] Louie J , Guthrie K . Strategies for assessing policy change efforts: a prospective approach. The Evaluation Exchange 2007;13(1):5.

[5] Anderson A . An introduction to theory of change. The Evaluation Exchange 2005;11(2):12, 19.

[6] McLaughlin JA , Jordan GB . Using logic models. In: Wholey JS, Hatry HP, Newcomber KE, editors. Handbook of practical program evaluation, San Francisco (CA): Jossey-Bass; 2010. p. 55–80.

[7] Adedokun OA , Childress AL , Burgess WD . Testing conceptual frameworks of nonexperimental program evaluation designs using structural equation modeling. Am J Eval 2011;32(4):480–93. 10.1177/1098214011401368

[8] Frieden TR , Dietz W , Collins J . Reducing childhood obesity through policy change: acting now to prevent obesity. Health Aff (Millwood) 2010;29(3):357–63. 10.1377/hlthaff.2010.0039 20194973

[9] Institute of Medicine. Accelerating progress in obesity prevention: solving the weight of the nation. Washington (DC): The National Academies Press; 2012.

[10] Glasgow L , Adams E , Joshi S , Curry L , Schmitt CL , Rogers T , Using a theory of change to guide grant monitoring and grantmaking. J Public Health Manag Pract 2017;23(2):126–30. 10.1097/PHH.0000000000000421 27598704

[11] Patton MQ . Utilization-focused evaluation. 4th edition. Thousand Oaks (CA): Sage; 2008.

[12] Campbell M , Coffman J . Tools to support public policy grantmaking. The Foundation Review 2009;1(3):123–31. 10.4087/FOUNDATIONREVIEW-D-09-00027.1

[13] Coffman J . A user’s guide to advocacy evaluation planning. Cambridge (MA): Harvard Family Research Project; 2009.

[14] Glantz K , Rimer BK , Lewis FM , editors. Health behavior and health education: theory, research and practice. 3rd edition. San Francisco (CA): Jossey-Bass; 2002.

[15] Niederdeppe J , Porticella N , Shapiro MA . Using theory to identify beliefs associated with support for policies to raise the price of high-fat and high-sugar foods. J Health Commun 2012;17(1):90–104. 10.1080/10810730.2011.585694 22059780

[16] Weiner B . On sin versus sickness. A theory of perceived responsibility and social motivation. Am Psychol 1993;48(9):957–65. 10.1037/0003-066X.48.9.957 8214914

[17] Blumberg SJ , Luke JV . Wireless substitution: early release of estimates from the National Health Interview Survey, July–December 2014. Atlanta (GA): US Department of Health and Human Services, Centers for Disease Control and Prevention, National Center for Health Statistics; 2015.

[18] American Association for Public Opinion Research. Standard definitions. Final dispositions of case codes and outcome rates for surveys. Oakbrook Terrace, (IL): American Association for Public Opinion Research; 2015.

[19] Schmitt CL , Juster HR , Dench D , Willett J , Curry LE . Public and policy maker support for point-of-sale tobacco policies in New York. Am J Health Promot 2014;28(3):175–80. 10.4278/ajhp.121023-QUAN-514 23875981

[20] Chromy JR . Sequential sample selection methods. In: Proceedings of the American Statistical Association, Section on Survey Research Methods. Alexandria (VA): American Statistical Association; 1979. p. 401–6.

[21] Howard KA , Rogers T , Howard-Pitney B , Flora JA , Norman GJ , Ribisl KM . Opinion leaders’ support for tobacco control policies and participation in tobacco control activities. Am J Public Health 2000;90(8):1283–7. 10.2105/AJPH.90.8.1283 10937010 PMC1446349

[22] Ingram DD , Franco SJ . NCHS urban–rural classification scheme for counties. Vital Health Stat 2 2012;2(154):1–65. 22783637

[23] Hancock GR , Mueller RO . Rethinking construct reliability within latent variable systems. In: Cudeck R, du Toit S, Sörbom D, editors. Structural equation modeling: present and future — a festschrift in honor of Karl Jöreskog. Lincolnwood (IL): Scientific Software International, Inc; 2001. p. 195–216.

[24] Thompson T , Benz J , Agiesta J , Brewer KH , Bye L , Reimer R , Junius D . Obesity in the United States: public perceptions. The Associated Press and NORC. 2013. http://www.apnorc.org/pdfs/obesity/ap-norc-obesity-research-highlights.pdf. Accessed April 10, 2018.

[25] Steiger JH . Understanding the limitations of global fit assessment in structural equation modeling. Pers Individ Dif 2007;42(5):893–8. 10.1016/j.paid.2006.09.017

[26] Tabachnick BG , Fidell LS . Using multivariate statistics. 5th edition. Boston (MA): Pearson; 2006.

[27] Comrey AL , Lee HB . A first course in factor analysis. 2nd edition. New York (NY): Psychology Press, 1992.

[28] Daynard RA . Lessons from tobacco control for the obesity control movement. J Public Health Policy 2003;24(3–4):291–5. 10.2307/3343375 15015862

[29] Sparks CH . Advocacy as a tobacco control strategy. In: Institute of Medicine. Ending the tobacco problem: a blueprint for the nation. Washington (DC): The National Academies Press; 2007. p. 690–703.

[30] Heinrich KM , Stephen MO , Vaughan KB , Kellogg M . Kansas legislators prioritize obesity but overlook nutrition and physical activity issues. J Public Health Manag Pract 2013;19(2):139–45. 10.1097/PHH.0b013e318254cc57 23358292

[31] Trochim WM . An introduction to concept mapping for planning and evaluation. Eval Program Plann 1989;12(1):1–16. 10.1016/0149-7189(89)90016-5

[32] Fishbein M , Ajzen I . Predicting and changing behavior: the reasoned action approach. New York (NY): Taylor and Francis Group; 2010.

[33] Gollust SE , Niederdeppe J , Barry CL . Framing the consequences of childhood obesity to increase public support for obesity prevention policy. Am J Public Health 2013;103(11):e96–102. 10.2105/AJPH.2013.301271 24028237 PMC3828688

[34] Schmitt CL , Allen JA , Kosa KM , Curry LE . Support for a ban on tobacco powerwalls and other point-of-sale displays: findings from focus groups. Health Educ Res 2015;30(1):98–106. 10.1093/her/cyu046 25096065

